# Notes

**Published:** 1895-07

**Authors:** 


### Notes.

The American Medical Publishers' Association held its second meeting in Baltimore, May 6 and 7. The following officers were unanimously re-elected: President, Dr. Landon B. Edwards, Richmond, Va.; Vice-President, Dr. J. C. Culbertson, Cincinnati, O.; Treasurer, J. McDonald, Jr., New York City; Secretary, Chas. Wood Fassett, St. Joseph, Mo. The next meeting will be held at Atlanta, Ga., 1896. Number 1 of the *Bulletin* has been issued and will be published monthly.

The third International Congress of Physiologists will meet at Berne, Switzerland, September 9th to 13th, 1895. The present officers are: President, Prof. Kronecker of Berne; C. S. Sherrington of London, general secretary for the English language; American secretary, Frederick S. Lee, Columbia College, N. Y. The languages recognized as official at the congress are English, French and German.

Sir Benjamin Richardson thinks that the normal period of human life is 110 years, and that seven out of ten average people ought to live that long, if they took proper care of themselves.

The epidermis of a brunette is said to be one-tenth of a millimeter thicker than that of a blond.

The most remarkable instance of rapid growth is said to be recorded by the French Academy in 1729. It was a boy six years of age, five feet, six inches in height. At the age of five his voice changed; at six his beard had grown, and he appeared a man of thirty. He possessed great physical strength, and could easily lift to his shoulders and carry bags of grain weighing 200 pounds. His decline was as rapid as his growth. At eight his hair and beard were gray; at ten he tottered in his walk, his teeth fell out, and his hands became palsied; at twelve he died with every outward sign of extreme old age.

"Tips."—Dr. Cocksedge, of Wales (*Medical Press*), places the following tips at the disposal of his brethren: If you have a fatiguingly deaf patient to talk to, place the ear pieces of your binaural stethoscope in the patient's ears and talk into the chest piece, and you have an excellent ear trumpet. If you leave your spectacles at home, being old and presbyopic, make a hole with a pin in the corner of your visiting card, and you can read your clinical thermometer or anything else.—*Exchange.*

Gallant Action of an Army Surgeon at Chitral.—Surgeon-Captain H. F. Whitchurch, attached to the 24th Bengal Native Infantry, performed a feat of exceptional gallantry on the occasion of the reconnaissance of March 3d from Fort Chitral. Captain Baird fell mortally wounded, but Surgeon-Captain Whitchurch, although running serious danger of death or capture by the enemy, would not abandon him, but, carrying the wounded officer on his back, fought his way back to the fort. —*Medical Press and Circular.*

*Teratologia.*—Such is the title of a quarterly journal recently instituted by Doctor J. W. Ballantyne, of Edinburgh, Scotland, and published by Williams & Norgate, 20 South Frederick street, same city. As its name indicates, it deals with congenital maladies and antenatal pathology—manifestly a large field. Not the least of its advantages is the complete review, given in each number, of current literature bearing upon teratological topics.—*Medical Age.*

A man recently sued a company for selling him a number of bottles of hair tonic "warranted to bring out the hair." In his papers the plaintiff says he was only partly bald when he began to use the tonic, and now he has not a single hair on his head. The defendants put in the plea that this is what they warranted to do: "To make the hair come out."—*National Medical Review.*

After the Bath.—According to *Le Mercredi Medical,* Max Edel, a German bacteriologist, took a bath and then examined the water for microbes; he found that it contained 5,850,-000,000! After a bath of one foot only, he estimated the number of microbes at 180,000,000.

The question now arises: When did Edel have his last previous bath?—*Medical Record.*

A recent writer calls attention to the fact that the failure of injection of morphine to produce sleep is often due to the fact that perfect silence is not maintained in the room where the patient is lying. Morphine tends to exalt the excitability, and for this reason a slight noise is liable to disturb the patient.

Dr. Hausman, an assistant of Prof. Virchow, declares that he has used the antitoxine of M. Roux and Herr Behring on proved cases of genuine diphtheria, without a particle of benefit and much harm.—*Exchange.*

Professor Osler, of Johns Hopkins, says that peneumonia is a self-limited disease, is not influenced by medicines, and there are no means at our command whereby it can be shortened in its course.

When patients rebel against large doses of iodide of potassium, these are easily borne by the stomach if the salt be mixed with soda-water instead of ordinary water.—*Practical Medicine.*

It is estimated that 293 hairs on the head, 39 on the chin, 23 on the forearm and 19 on the back of the hand are respectively contained in an area of a quarter of an inch.

According to the *Medical Record* castor oil has not failed in any case to remove warts when applied once a day for two to six weeks.

A Philadelphia oculist who has been studying the human eye for thirty years, declares that all great men of the past and present had or have blue or gray eyes.

